# Quantitative analysis of cornea endothelial cell damage from enucleation, corneal buttoning, and storage in donor corneas using trypan blue dye staining

**DOI:** 10.1097/MD.0000000000030430

**Published:** 2022-09-09

**Authors:** Young Chae Yoon, Yong-Soo Byun, Patrick Kim, Min Ji Ha, Woong Joo Whang, Kyung Sun Na, Eun Chul Kim, Hyun Seung Kim, Ho Sik Hwang

**Affiliations:** a Department of Ophthalmology, College of Medicine, The Catholic University of Korea, Seoul, Korea; b Department of Ophthalmology, Chuncheon Sacred Heart Hospital, Hallym University College of Medicine, Chuncheon, Korea.

**Keywords:** buttoning, cornea endothelial cell damage, corneadonor cornea, corneatrypan blue dye staining

## Abstract

We aimed to quantitatively analyze the corneal endothelial cell damage by measuring the area stained with trypan blue dye, and to confirm the degree of corneal endothelial cell damage resulting from enucleation, corneal buttoning, and storage in donor corneas intended for use in human corneal transplantation. This study was a retrospective analysis of medical records and videos recorded during keratoplasty. Twenty-one corneal buttons of 21 donors that underwent endothelial cell staining using trypan blue for the donor preparation during DALK or DMEK were included in the study. The percentage of stained area in entire corneal endothelia and the percentage of the stained area in the 8-mm diameter circle were quantitatively analyzed using Adobe Photoshop. The mean percentage of the stained area in the entire corneal endothelia in 13 corneas was 8.1 ± 13.3% (range, 0.0–56.1%), and the mean percentage of the stained area in a circle with a diameter of 8 mm in 21 corneas was 3.4 ± 5.2% (range, 0.0–18.9%). The correlations between the death-to-preservation time, the training duration of the residents who performed donor corneal buttoning, and the percentage of the stained area in the 8-mm diameter circle were not significant(*P* = .441, *P* = .495, respectively). Cornea thickness and endothelial cell density did not differ between 10 eyes in the group with the percentage of the stained area in a circle with a diameter of 8 mm <5% and 5 eyes in the group with the percentage more than 5% damage (*P* = .854, *P* = .358). The corneal endothelial cell damage could be quantitatively analyzed using trypan blue staining before keratoplasty in donor cornea. The amount of corneal endothelial cell damage in the central 8-mm circle was mostly acceptable, but some cases showed significantly severe endothelial cell damage. The corneal thickness and endothelial cell density did not differ between 10 eyes in the group with the percentage of the stained area in a circle with a diameter of 8 mm <5% and 5 eyes in the group with the percentage more than 5% damage. Therefore, pachymetry and specular microscopy are not sufficient for evaluating donor corneas before keratoplasty.

## 1. Introduction

Donor corneas are generally evaluated using slit-lamp microscopy, pachymetry, and specular microscopy. Among these tests, specular microscopy is one of the most important tests because endothelial cell density is used as a criterion for the suitability of the donor cornea for keratoplasty. Specular microscopy, however, has some limitations for donor cornea evaluation. First, it measures the corneal endothelial cell density in only a very limited area of the cornea.^[1]^ For example, the Konan Kerato Analyser EKA-98 (Konan Medical Inc., Hyogo, Japan) specular microscope measures endothelial cell density in only a 0.2-mm × 0.4-mm area. Specular microscopy for donor corneas is usually performed after enucleation, corneal buttoning, and storage. Because the endothelial damage resulting from these processes is not usually uniform across the entire endothelial surface, the endothelial cell density in a limited area is not adequately representative of the entire endothelial surface. Second, specular microscopy does not distinguish cells that are dead or have degenerative changes from healthy viable cells.^[2,3]^ Therefore, we hypothesized that pachymetry and specular microscopy are not sufficient for evaluating donor corneas prior to keratoplasty. These limitations of pachymetry and specular microscopy raise the need for a simple anatomic evaluation method that is objective, accurate, and allows for assessment of the entire endothelial surface.

One method for anatomically evaluating corneal endothelial damage is to estimate the percentage of damaged corneal endothelia by measuring the area stained by a vital staining solution that selectively stains damaged corneal endothelial cells. In 1981, Taylor and Hunt^[4]^ first attempted to visualize and quantify damaged corneal endothelial cells using the vital stain trypan blue and intercellular stain Alizarin red S staining. They stained the corneas of rabbits and pigs, as well as 1 human cornea, and concluded that evaluating corneal endothelial integrity using these 2 stains provided a simple and quick technique for visualizing both damaged and normal cells, thereby permitting quantification of endothelial cell damage. Since their report, many researchers have reported the degree of corneal endothelial cell damage from experimental donor punches, donor preparations, and lamellar keratoplasty procedures using various corneal staining solutions for donor corneas not suitable for keratoplasty in patients.^[5–10]^

In our hospitals, we use trypan blue staining for donor preparation during Descemet membrane peeling in the course of deep anterior lamellar keratoplasty (DALK) or Descemet membrane endothelial keratoplasty (DMEK). In this process, we observed that corneal endothelial cell damage resulting from enucleation, storage, and corneal buttoning varies considerably from cornea to cornea. Therefore, in the present study, we quantitatively analyzed the corneal endothelial cell damage resulting from enucleation, corneal buttoning, and storage in donor corneas intended for use in human corneal transplantation. Further, correlation analysis or comparison were performed to determine whether the percentage of corneal endothelial cell damage was related to the death-to-preservation time, the training duration of the residents who performed the donor eyeball enucleation and corneal buttoning, central corneal thickness, and preoperative endothelial cell density or not.

## 2. Patients and Methods

This study followed the principles of the Declaration of Helsinki and was approved by the Institutional Review Board (IRB) of Yeouido St. Mary’s Hospital of the Catholic University of Medicine (IRB approval number: SC20RIDI0065), Seoul St. Mary’s Hospital of the Catholic University of Medicine (IRB approval number: KC20RIDI0420), and Chuncheon Sacred Heart Hospital of the Hallym University College of Medicine (IRB approval number: 2020-05-003). This study was a retrospective analysis of medical records and videos recorded during keratoplasty.

Twenty-one corneal buttons of 21 donors that underwent endothelial cell staining using trypan blue for the donor preparation during DALK or DMEK performed at Yeouido St. Mary’s Hospital, Seoul St. Mary’s Hospital, and Chuncheon Sacred Heart Hospital from January 2015 to May 2020, were included in the study. Six donor corneas were used for DMEK. Fifteen donor corneas were used for DALK. The corneal endothelium and Descemet membranes separated from 4 donor corneas during DALK were used for DMEK in another patients.

All corneas included in this study were donated from Korean donors. All donor corneas were buttoned according to standard techniques^[11]^ after enucleation. The enucleation and buttoning were performed by residents training in ophthalmology. The sclerocorneal buttons were immediately placed in corneal storage medium (Optisol®, Chiron Ophthalmics, Irvine, CA) and stored at 4°C until surgery. All corneas included in the study were from donors with no intraocular tumor or systemic infectious disease, and the corneal endothelial cell density measured by specular microscopy was greater than 2000 cells/mm^2^. Therefore, all corneas included in the study were available for corneal transplantation in patients.

Through medical record review, the age and sex of the donors, death-to-preservation time, central corneal thickness, and corneal endothelial cell density were evaluated. The training duration of the ophthalmology residents who performed the donor corneal buttoning was also investigated. The central corneal thickness of the donor corneas used at Yeouido St. Mary’s Hospital and Seoul St. Mary’s Hospital was measured by ultrasound pachymetry (Dicon P55, Paradigm Medical Industries Inc., Salt Lake City, UT) of the eyeball. The corneal endothelial cell density was measured using a noncontact eye-bank specular microscope (Konan Kerato Analyser EKA-98, Konan Medical Inc., Hyogo, Japan) at the center of the cornea after corneal buttoning. For donor corneas used at Chuncheon Sacred Heart Hospital, the central corneal thickness was measured using the Spectralis optical coherence tomography (OCT) (Heidelberg engineering, Heidelberg, Germany), and a noncontact specular microscope (Specular microscope, Noncon Robo-CA, Konan Medical Inc., Hyogo, Japan) was used to measure the corneal endothelial cell density.

### 2.1. Staining and photographic procedures

The surgeries, including donor preparation, were recorded by a video camera attached to an ophthalmic surgical microscope. An ophthalmic surgical microscope (Leica M844 F40, Leica Microsystems GmbH, Wetzlar, Germany) was used at Yeouido St. Mary’s Hospital and Chuncheon Sacred Heart Hospital, and an OPMI Lumera® T (Carl Zeiss Meditec, Inc., Dublin, CA) was used at Seoul St. Mary’s Hospital. The sclerocorneal button was removed from the corneal storage medium and placed on a donor punch block with the corneal endothelium facing upward. Next, 0.06% trypan blue (Vision Blue®, DORC International, Zuidland, The Netherlands) was dropped onto the corneal endothelium to stain the damaged corneal endothelia. After 20 to 30 seconds, drops of balanced salt solution were applied to the corneal endothelium to gently wash off the remaining trypan blue solution. In 3 eyes, the trypan blue staining was performed before scoring with a Sinskey hook. In 10 eyes, the trypan blue staining was performed after partial trephination for Descemet membrane peeling. In 8 eyes, the trypan blue staining was performed after scoring using a Sinskey hook for Descemet membrane peeling. We captured the best images of the stained corneal endothelia from the recorded videos.

### 2.2. Quantitative analysis

**Figure 1.**
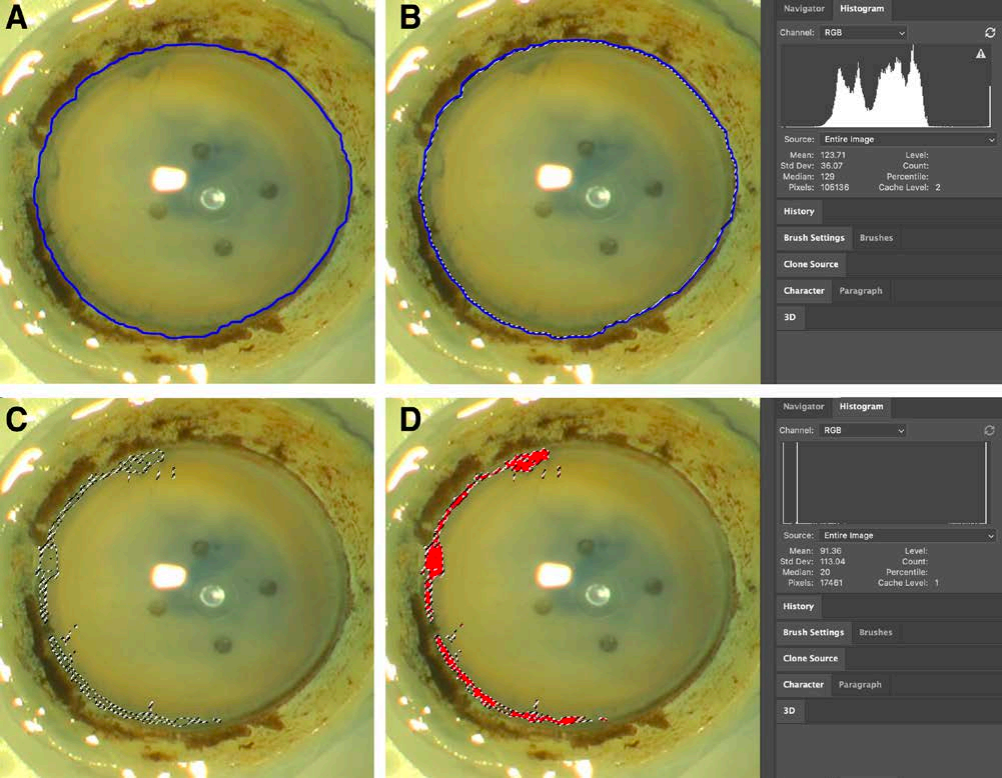
How to get the stained area in entire corneal endothelia using Adobe photoshop 2020. (A) By using the “Magnetic Lasso Tool”, the circumference of the corneal graft tagging along with the Schwalbe line was selected. The area within the blue line is the selected area. (B) The number of pixels making up the selected area was calculated using the “image/histogram function”. (C) Next, returning to the original photograph, the stained area was precisely selected using the “magic wand tool”. (D) The area filled with red color is the selected area. The number of pixels stained with trypan blue was calculated using the “image/histogram function”. The percentage of the stained area in the entire corneal endothelia was obtained by dividing the pixel number of the trypan blue stained area by the pixel number of the entire corneal endothelia. (A) Each photograph of the entire corneal endothelia was opened in the software program Adobe Photoshop 2020, and the entire corneal endothelial surface was selected along the Schwalbe line using the “magnetic lasso tool”, (B) The number of pixels making up the selected area was calculated using the “image/histogram function”, (C) Next, returning to the original photograph, the stained area was precisely selected using the “magic wand tool”, (D) The number of pixels stained with trypan blue was calculated using the “image/histogram function”. The percentage of the stained area in the entire corneal endothelia was obtained by dividing the pixel number of the trypan blue stained area by the pixel number of the entire corneal endothelia (Fig. 1-D).

All processes used to determine the percentage of stained endothelia were performed by 1 researcher (Y.C.Y.) with Adobe Photoshop 2020 (Adobe Systems, San Jose, CA). Each photograph of the entire corneal endothelia was opened in the software program Adobe Photoshop 2020, and the entire corneal endothelial surface was selected along the Schwalbe line using the “magnetic lasso tool” (Fig. 1-A). The number of pixels making up the selected area was calculated using the “image/histogram function” (Fig. 1-B). Next, returning to the original photograph, the stained area was precisely selected using the “magic wand tool” (Fig. 1-C). The number of pixels stained with trypan blue was calculated using the “image/histogram function”. The percentage of the stained area in the entire corneal endothelia was obtained by dividing the pixel number of the trypan blue stained area by the pixel number of the entire corneal endothelia (Fig. 1-D).

**Figure 2.**
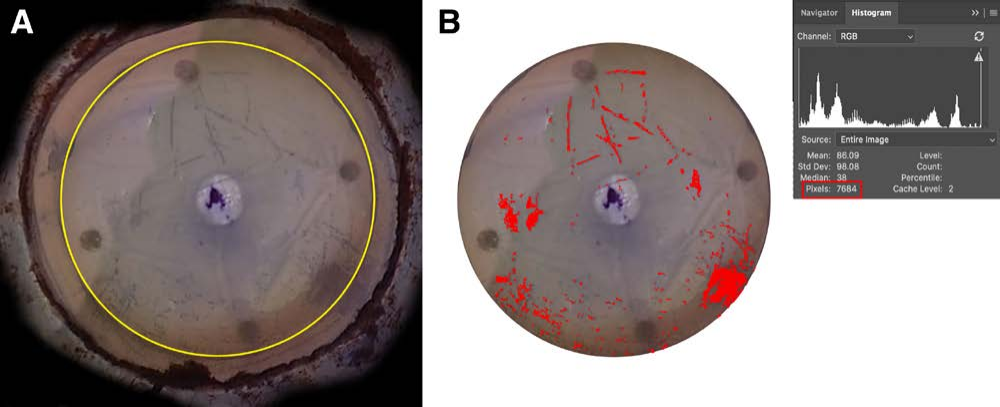
How to get the stained area in the 8-mm diameter circle using Adobe photoshop 2020. (A) The light blue line is a guideline that displays a range of 8mm from the center of the cornea, assuming that the end-to-end length of the Schwalbe line is 11.5 mm. Based on this light blue line, an 8-mm diameter circle was selected (yellow line). The 8-mm diameter circle in the center of the cornea was selected using the “circular marquee tool”, and the total pixel number of the selected area was obtained using the “image/histogram function” (B) The area stained with trypan blue within the 8-mm diameter circle was selected using the “magic wand tool”, and the “image/histogram function” was used to obtain the pixel number of the selected area. The area filled with red color is the selected area.

In many cases, it was difficult to determine the trypan blue staining of endothelial cells at the peripheral endothelial surface near the Schwalbe line. Therefore, we additionally calculated the percentage of the trypan blue stained area in a central 8-mm diameter circle. In most corneal transplantations, the diameter of the donor cornea is approximately 8 mm. Therefore, the percentage of the trypan blue stained area in an 8-mm diameter circle is more clinically relevant than that of the trypan blue stained area of the entire endothelial cell area. The mean corneal diameter from the Schwalbe line to the opposite Schwalbe line is assumed to be 11.5 mm in Koreans,^[12]^ so an 8-mm diameter circle was set in the center of the corneal endothelial surface in the captured image using a proportional equation. An 8-mm diameter circle in the center of the cornea was selected using the “circular marquee tool”, and the total pixel number of the selected area was obtained using the “image/histogram function” (Fig. 2-A). The area stained with trypan blue within the 8-mm diameter circle was selected using the “magic wand tool”, and the “image/histogram function” was used to obtain the pixel number of the selected area (Fig. 2-B). As in the previous method, the percentage of the stained area in the 8-mm diameter circle was obtained by dividing the pixel number of the trypan blue stained area by the pixel number of the 8-mm diameter circle.

**Figure 3.**
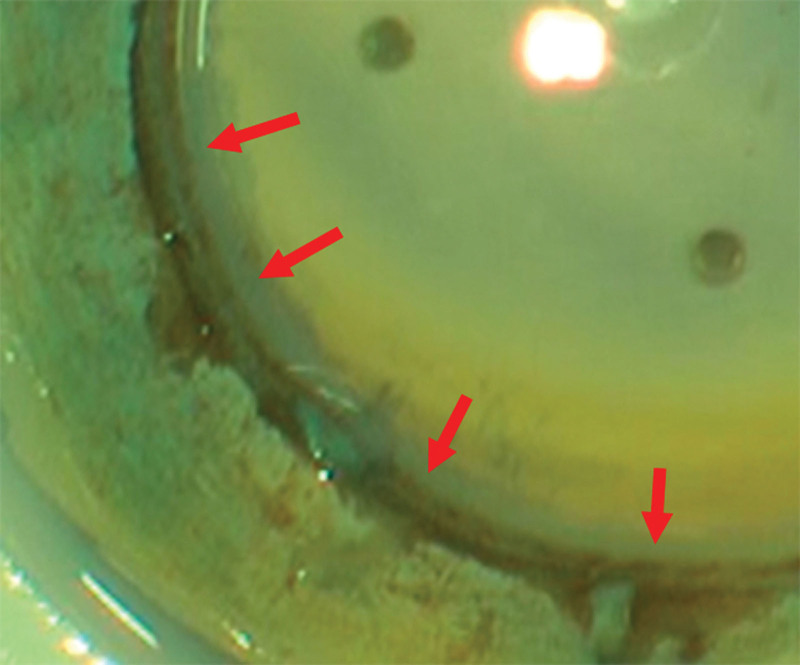
Ambiguous areas whether they were stained with trypan blue or not. This cornea was scored and then stained with trypan blue. It is not clear whether the pointed area with red arrows in this figure is stained with trypan blue or not.

Endothelial cell damage from scoring or partial trephination was not included in the trypan blue stained area because we wanted to investigate the endothelial cell damage resulting only from enucleation, corneal buttoning, and storage. In cases of partial trephination (10 eyes), the trypan blue stained area produced by partial trephination was clearly distinguishable, making it easy to exclude this area. In cases in which a Sinskey hook was used for scoring (8 eyes), it was often difficult to distinguish the trypan blue stained area induced by the scoring from the stained area caused by corneal buttoning (Fig. 3). Therefore, when calculating the percentage of the stained area in the entire corneal endothelia, these 8 eyes were excluded from the analyses. Because scoring is performed outside of the 8-mm diameter circle, however, these 8 eyes were included when analyzing the percentage of the stained area in an 8-mm diameter circle.

### 2.3. Statistics

The mean and standard deviation of donor age, death-to-preservation time, training duration of the residents who performed the donor corneal buttoning procedure, central corneal thickness, corneal endothelial cell density, percentage of the stained area in the entire endothelial surface, and the percentage of the stained area in a circle with a diameter of 8 mm were obtained. Spearman rank-order correlation analysis, a nonparametric analysis, was used to evaluate differences between the percentage of the stained area in an 8-mm diameter circle and death-to-preservation time and training duration of the residents who performed the donor corneal buttoning procedure. The central corneal thickness and corneal endothelial cell density measured using a specular microscope were compared (Mann-Whitney U-test) in the 2 groups, divided into a group with the percentage of the stained area in a circle with a diameter of 8 mm <5% and a group with the percentage of 5% or more. The percentage of the stained area in an 8-mm diameter circle was significantly higher in 3 eyes compared with the other eyes, and therefore the Mann-Whitney test was used to compare the mean training duration of the residents who performed the donor corneal buttoning procedure for these 3 eyes and the mean training duration of residents who performed the donor corneal buttoning procedure for the remaining eyes. For statistical analysis, SPSS version 22.0 (SPSS Inc., Chicago, IL) was used and *P* < .05 was considered statistically significant.

## 3. Results

**Figure 4.**
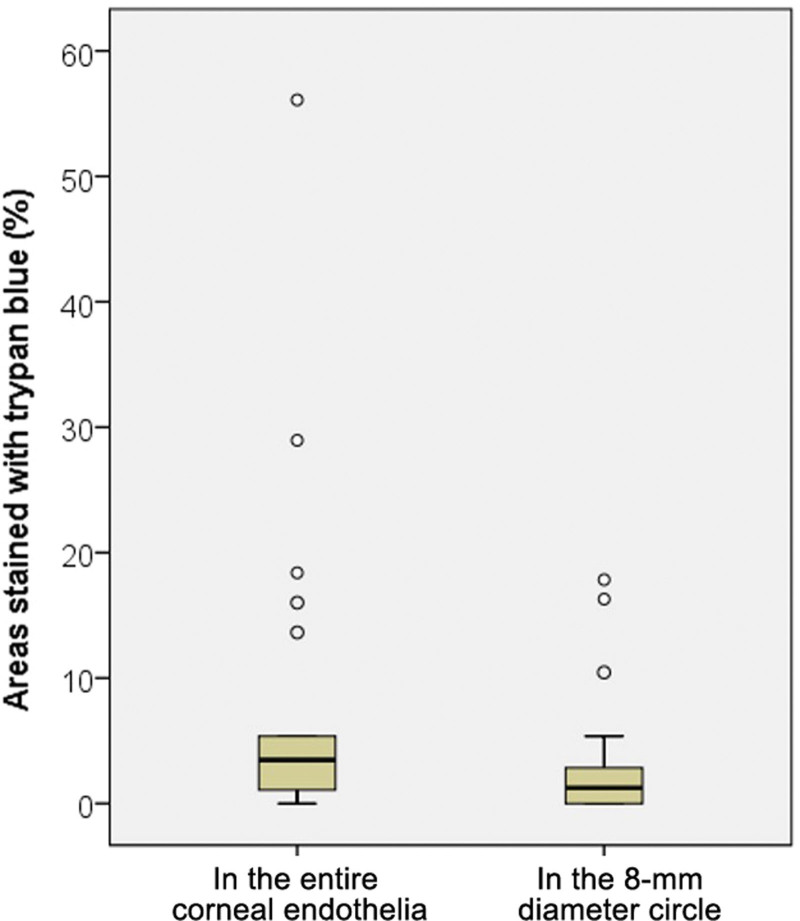
The box and whisker plots for the distribution of trypan blue stained area in the entire corneal endothelia and in the 8-mm diameter circle. The boxes denoted interquartile range (IQR, 25^th^-75^th^ percentile) of stained area. The whiskers denoted range of the trypan blue stained area, and dots denoted outliers. A horizontal line in the box denoted median of trypan blue stained area, each. The mean percentage of the stained area in the entire corneal endothelia in 13 corneas was 8.1 ± 13.3% (range, 0.0–56.1%), and the mean percentage of the stained area in the 8-mm diameter circle in 21 corneas was 3.4 ± 5.2% (range, 0.0–18.9%).

A total of 21 eyes were included in the study. Six of the eyes had no donor information or donor cornea, so only the percentage of trypan blue staining in the images were obtained for these eyes. The mean age of the corneal donors was 57.5 years (7 men, 8 women). The mean death-to-preservation time was 13.1 hours. The mean training duration of the residents who performed the donor corneal buttoning was 18.4 months. The mean central corneal thickness was 587 µm and the mean corneal endothelial cell density measured by a noncontact specular microscope was 2812 cells/mm^2^ (Table 1). The mean percentage of the stained area in the entire corneal endothelia in 13 corneas was 8.1 ± 13.3% (range, 0.0–56.1%), and the mean percentage of the stained area in the 8-mm diameter circle in 21 corneas was 3.4 ± 5.2% (range, 0.0–18.9%) (Table 1, Fig. 4).

**Table 1:** Demographics of cornea donors and characteristics of donor corneas included in the study.

	Mean	Range
Number of donors (n)	21	
Number of eyes (n)	21	
Age (years) (n = 15)*	57.5 ± 9.6	36.0–72.0
Sex (men:women) (n = 15)*	7: 8	
Death-to-preservation time (hours) (n = 15)*	13.1 ± 7.9	2.6–33.5
Training duration of the residents who performed donor corneal buttoning (months) (n = 15)*	18.4 ± 7.1	5.0–29.0
Central corneal thickness (µm) (n = 15)*	587 ± 77	356–696
Endothelial cell density (cells/mm^2^) (n = 15)*	2812 ± 356	2142-3436
Stained area in entire corneal endothelia (%) (n = 13)†	8.1 ± 13.3	0.0–56.1
Stained area in an 8-mm diameter circle (%) (n = 21)	3.4 ± 5.2	0.0–18.9

Values are presented as mean ± standard deviation unless otherwise indicated.

* In 6 eyes, the donor information could not be obtained from the medical records and they were not included in the statistics.

† In 8 eyes, there were many areas in which it was difficult to determine whether or not they were trypan blue stained because they were scored before the trypan blue dying; these eyes were not included in the statistical analyses.

**Figure 5.**
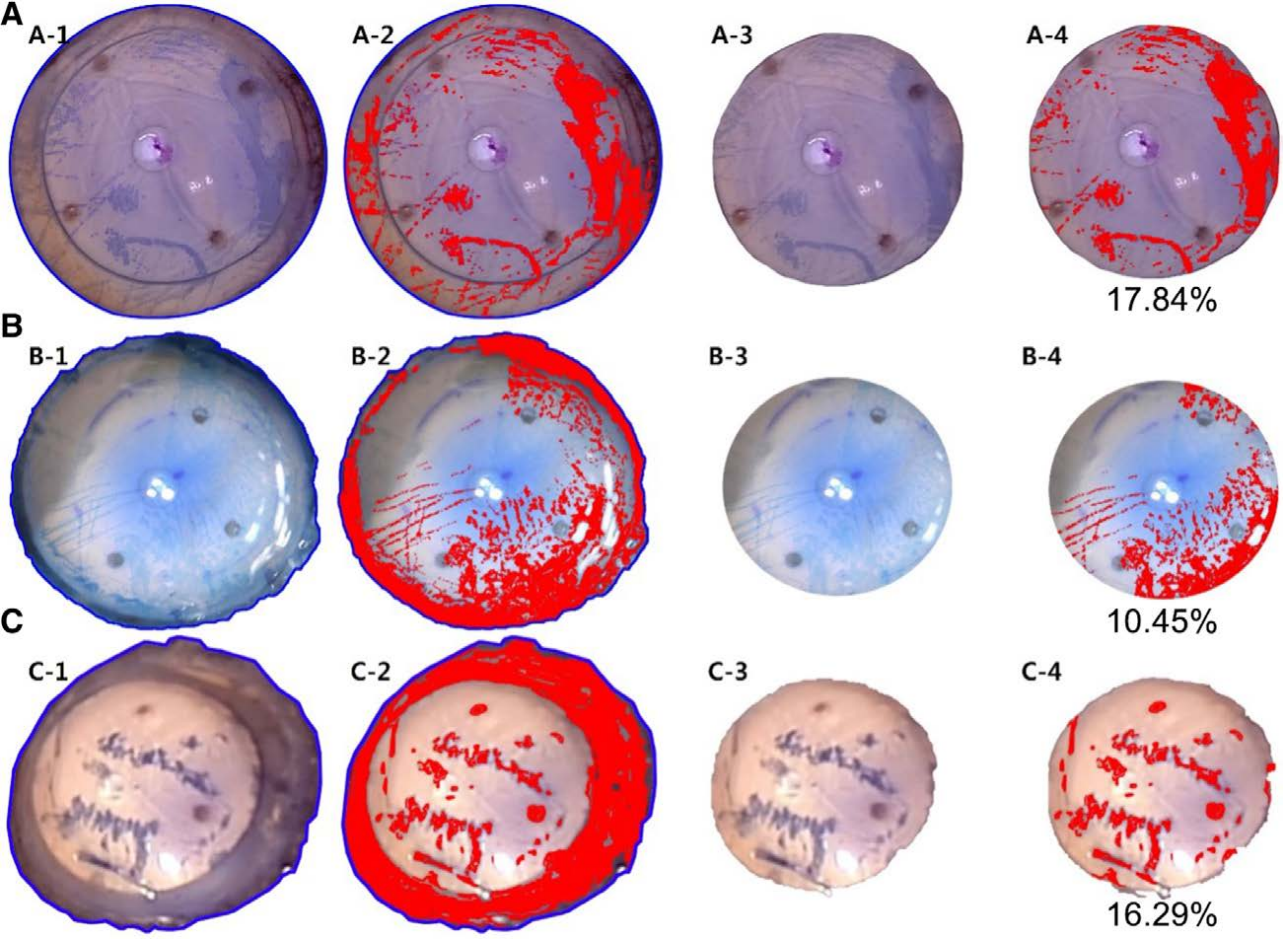
The outliers in trypan blue staining. The percentage of the stained area in an 8-mm diameter circle in 3 donor corneas (A, B, C) was 17.8%, 10.5%, and 16.3%, significantly higher than that of the other donor corneas. The blue lines in (A-1), (B-1) and (C-1) means the circumference of the corneal graft tagging along with the Schwalbe line. The red colored area in (A-2), (B-2), and (C-2) means the trypan blue stained area of (A-1), (B-1), and (C-1), respectively. (A-3), (B-3), and (C-3) shows the 8-mm diameter circle. (A-4), (B-4), and (C-4) shows the trypan blue stained area in the 8-mm diameter circle by red color.

**Figure 6.**
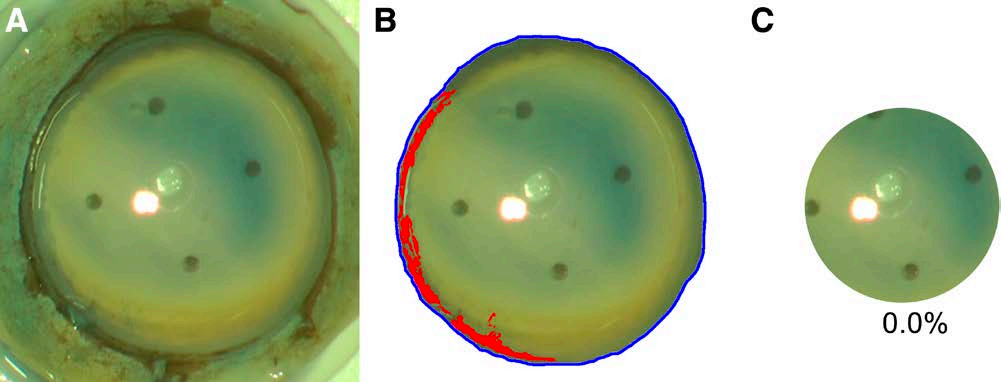
A cornea rarely stained with trypan blue. (A) This figure showed trypan blue staining percentage of 0% in the 8-mm diameter circle, and unlike the images of Figure 5. It was rarely stained. (B) This figure shows the blue line which is the circumference of the corneal graft tagging along with the Schwalbe line, and trypan blue stained area inside the blue line. The trypan blue stained area was painted by red color to make it more visible. (C) This figure shows the area in the 8-mm diameter circle. The percentage of stained area in the 8-mm diameter circle was 0 % in this cornea.

The percentage of the stained area in an 8-mm diameter circle in 3 donor corneas (Fig. 5) was 17.8%, 10.5%, and 16.3%, significantly higher than that of the other donor corneas (Fig. 6). Figure 5A (17.8%) shows a captured image from DALK donor preparation video. The stroma of this cornea was used for DALK, and the peeled Descemet membrane and endothelium were transplanted by DMEK to another patient with Fuchs’ dystrophy. The Descemet membrane was not manipulated excessively during DMEK in the anterior chamber. The Descemet membrane was not detached after surgery, so rebubbling was not required. The corneal endothelial cell density at 2 months postoperatively, however, was only 667 cells/mm^2^, perhaps because of the high corneal endothelial damage in an 8-mm diameter circle (17.8%). The donor cornea shown in Figure 5-B (10.5% damage within the 8-mm circle) was also used for DALK, and it was not known whether the peeled Descemet membrane and endothelium were used for DMEK in another patient because that information was not recorded. The donor cornea shown in Figure 5-C (16.3% damage within the 8-mm circle) was also used for DALK. The surgeon recognized significant endothelial damage during donor preparation and decided not to use the peeled Descemet membrane and endothelia for DMEK in another patient. In these 3 cases (Fig. 5-A–C), the mean training duration (17.0 ± 4.4 months) of the residents who performed the donor corneal buttoning was not significantly different from the mean training duration (18.8 ± 7.7 months) of the residents who performed the donor corneal buttoning of the remaining 12 eyes (Mann-Whitney test, *P* = .734).

**Figure 7.**
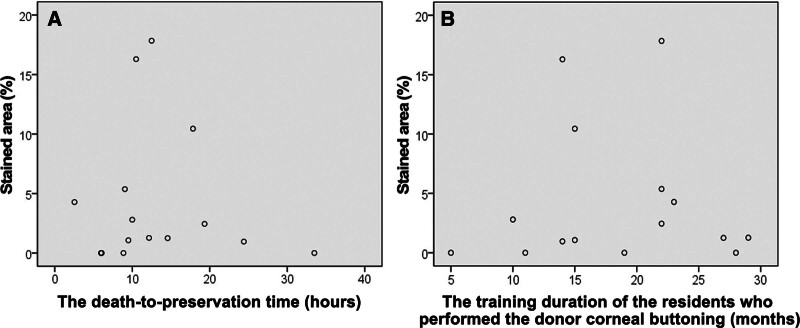
The scatters plots of the percentage of the stained area in the 8-mm diameter circle (%) and ocular parameters. (A) The percentage of the stained area in an 8-mm diameter circle did not significantly correlate with the death-to-preservation time (Spearman Rho, *R* = 0.215, *P* = .441). (B) The percentage of the stained area in an 8-mm diameter circle did not significantly correlate with the training duration of the residents who performed the donor corneal buttoning (Spearman Rho, *R* = 0.191, *P* = .495).

The percentage of the stained area in an 8-mm diameter circle did not significantly correlate with the death-to-preservation time (Spearman Rho, *R* = 0.215, *P* = .441; Fig. 7-A). The percentage of the stained area in an 8-mm diameter circle did not significantly correlate with the training duration of the residents who performed the donor corneal buttoning (Spearman Rho, *R* = 0.191, *P* = .495; Fig. 7-B).

**Figure 8.**
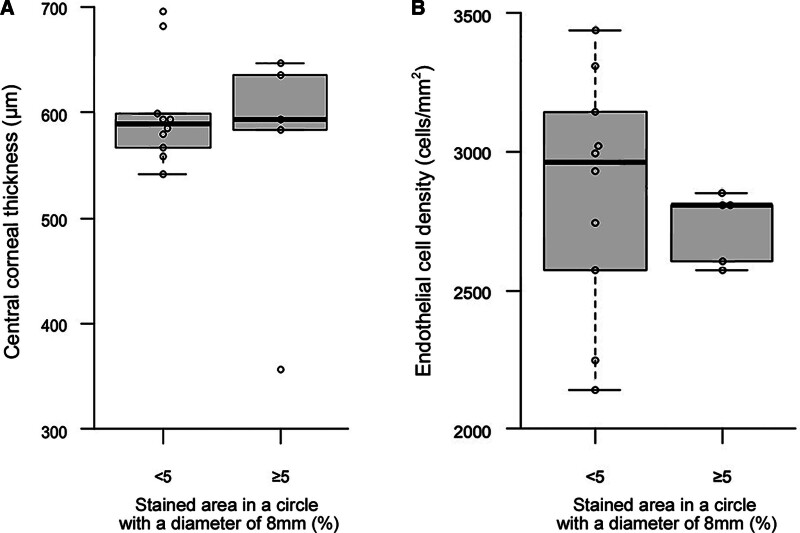
Comparison of cornea thickness and endothelial cell density measured using a specular microscope between 10 eyes in the group with the percentage of the stained area in a circle with a diameter of 8 mm <5% and 5 eyes in the group with the percentage more than 5% damage. Cornea thickness (A) and endothelial cell density (B) did not differ between the 2 groups (Mann-Whitney u-test, *P* = .854, *P* = .358, respectively).

Cornea thickness did not differ between 10 eyes in the group with the percentage of the stained area in a circle with a diameter of 8 mm <5% and 5 eyes in the group with the percentage more than 5% damage (Mann-Whitney U-test, *P* = .854)(Fig. 8A). Group sample sizes of 10 and 5 achieve 63.652% power to reject the null hypothesis of equal means when the population mean difference is μ1 − μ2 = 599.0 − 749.0 = −150.0 with a standard deviation for both groups of 118.6 and with a significance level (alpha) of 0.050 using a 2-sided 2-sample equal-variance z-test.

Corneal endothelial cell density measured using a specular microscope did not differ between 10 eyes in the group with the percentage of the stained area in a circle with a diameter of 8 mm <5% and 5 eyes in the group with the percentage more than 5% damage (Mann-Whitney U-test, *P* = .358)(Figure 8B). Group sample sizes of 10 and 5 achieve 89.239% power to reject the null hypothesis of equal means when the population mean difference is μ1 − μ2 = 2854.0 − 2104.0 = 750.0 with a standard deviation for both groups of 428.0 and with a significance level (alpha) of 0.050 using a 2-sided 2-sample equal-variance z-test.

## 4. Discussion and Conclusions

Some researchers have reported the degree of corneal endothelial cell damage from experimental donor punch, donor preparations, and lamellar keratoplasty procedures using various corneal staining solutions for donor corneas not suitable for keratoplasty in patients. Davis-Boozer et al^[5]^ reported immediate endothelial cell loss resulting from insertion of a precut donor button for Descemet stripping automated endothelial keratoplasty (DSaEK). Ten corneas were precut for DSaEK and trephinated to a diameter of 8.0 mm (n = 5) or 8.5 mm (n = 5). Each tissue was placed onto a spatula and inserted into a cadaveric whole globe through a 5.2-mm incision. The tissue was carefully removed and stained with trypan blue and Alizarin red S to detect damaged endothelium. They reported that the mean endothelial cell damage rate was 15.6% (95% confidence interval, 13.8–17.4). Griffin et al^[6]^ reported corneal endothelial cell damage from DMEK donor preparation using cadaver corneas with calcein AM dye staining. They photographed prestripped donor corneas with a digital camera and analyzed them after staining with either “‘Fiji’” image analysis software in the open-source “ImageJ’’ or Adobe Photoshop. The mean percentage of the stained area in an 8-mm diameter circle was 22.5 ± 6.5% with Fiji and 18.7 ± 7.0% with Adobe Photoshop (*P* = .04). Tran et al^[9]^ evaluated DMEK grafts to determine graft quality and feasibility of DMEK grafts that had been prestripped and preloaded into injectors by eye bank technicians before shipping to surgeons. They loaded the donor Descemet membrane peeled for DMEK into a Straiko Modified Jones tube in 31 eyes, and then quantitatively analyzed the extent of endothelial cell damage using calcein-AM staining and the Fiji program. The mean corneal endothelial cell damage rate was 16.8 ± 5.9% in the donor corneas previously loaded into a Jones tube and 9.3 ± 5.9% in non-preloaded corneas. Wholle et al^[10]^ evaluated the endothelial cell damage of preloaded DMEK grafts to determine the safety of preloaded DMEK grafts for 24 hours before surgery by analyzing endothelial cell loss. They loaded 18 corneas into a modified Jones’ tube that had been filled with Optisol. Nine eyes were stained with calcein AM 1 minute after loading, the other 9 eyes were stained with calcein AM 24 hours after loading. The early stained eyes had 19.2 ± 7.2% corneal endothelial damage and the 24-hour stained eyes had 22.0 ± 4.0% damage.

To our knowledge, there are no reports of quantitative analysis of the corneal endothelial damage after only enucleation, buttoning, and storage using trypan blue staining of donor corneas that were transplanted into patients. We inevitably had to stain the donor cornea with trypan blue during the donor preparation for DALK or DMEK. During donor preparation, we found that some corneas actually had a lot of endothelial damage induced by the enucleation, buttoning, and storage procedures. If we evaluated the donor corneal endothelia by only specular microscopy or pachymetry, we might have concluded that they were all good-quality donor corneas. If corneas with severe endothelial damage had been transplanted into patients, the corneal transplantation prognosis would have been poor. The peeled Descemet membrane and endothelium of the cornea shown in Fig. 5A (17.8% endothelial damage) was transplanted into another patient with Fuchs’ dystrophy by DMEK. Even without excessive manipulation of the Descemet membrane during DMEK and no rebubbling, the corneal endothelial cell density at 2 months postoperatively was only 667 cells/mm^2^. The central corneal thickness and endothelial cell density measured before corneal transplantation did not differ between 10 eyes in the group with the percentage of the stained area in a circle with a diameter of 8 mm <5% and 5 eyes in the group with the percentage more than 5% damage. Therefore, pachymetry and specular microscopy are not sufficient for evaluating donor corneas prior to keratoplasty. Recently, we use prestripped Descemet membrane for DMEK and do not peel Descemet membrane from donor cornea during DALK. Therefore, a sufficient number of corneas were not included in the study. For the hypothesis, statistical power for corneal thickness was low at 63.652%, but power for endothelial cell density was high at 89.23% in the study.

A question may be raised regarding whether trypan blue staining should be applied to all corneal transplantations, including penetrating keratoplasty. Trypan blue staining has the advantage of allowing investigators to visualize the endothelial cell damage in the donor cornea. Trypan blue staining itself, however, produces endothelial cell toxicity, and therefore minimizing the exposure time to the staining reagent minimizes the toxicity.^[13]^ Even if the stain-induced endothelial cell toxicity is minimal after a short exposure, trypan blue stain cannot be recommended for all corneal transplantations due to its toxicity.^[14]^ We suggest that trypan blue stain is not recommended for penetrating keratoplasty or DSaEK. Trypan blue stain of endothelium is not necessary for DALK. However, it is inevitable to use trypan blue stain during DMEK donor preparation. We have to check the trypan blue stained area in the entire corneal endothelia during DMEK donor preparation. If the area of the corneal endothelial cells stained with trypan blue is too large, it should not be used as a donor cornea. Ideally, the development of a vital staining reagent that can clearly stain damaged cells with low toxicity will be helpful for evaluating the health of donor corneal endothelial cells before keratoplasty.

In previous studies, the percentage of the corneal endothelial cell area stained with a vital staining solution in only an 8-mm diameter circle was measured.^[5–10]^ In the present study, unlike previous studies, we analyzed the percentage of corneal endothelial cell damage in not only an 8-mm diameter circle, but also the entire corneal endothelial surface. As a result, the mean percentage of corneal endothelial cell damage for the entire corneal endothelial surface was 8.1 ± 13.3% (range, 0.0–56.1%), but the mean percentage of corneal endothelial damage in the 8-mm diameter circle was 3.4 ± 5.2% (range, 0.0–18.9%), which is a big difference. Considering the usual donor size for keratoplasty is approximately 8 mm, the corneal endothelial damage in an 8-mm diameter circle is more clinically relevant than that for the entire corneal endothelial surface. We also calculated the percentage of corneal endothelial cell damage for the entire corneal endothelial surface, however, and found that the corneal endothelial damage from corneal buttoning was more common in the periphery than in the central 8-mm diameter circle.

The buttoning process is one of the most important processes in the preparation of donor cornea because it requires delicate handling and the amount of corneal endothelial cell damage induced depends on the skill of the person doing the buttoning. In some countries, not only the enucleation or buttoning, but also precutting of the donor cornea is performed by eye bank technicians. In Korea, ophthalmology residents in training perform eyeball enucleation and corneal buttoning. In this study, the corneal endothelial damage and the training duration of the residents who performed donor corneal buttoning were not significantly correlated.

As a retrospective study, endothelial damage might not have been effectively detected because we analyzed the still images by capturing previously recorded movie files. Because the cornea has a curvature, measuring the endothelial damage area from the captured photos differs from the actual corneal endothelial cell damage area. This error was not corrected in previous studies, and the difference may be smaller in the central 8 mm area, which is more clinically important in keratoplasty.

We did not use Alizarin red S solution in addition to the trypan blue solution for endothelial staining as in the previous studies.^[4,5,7]^ Trypan blue stains the nuclei of dead cells that do not have a plasma membrane. On the other hand, Alizarin red S stains the intercellular space or Descemet membrane of necrotic corneal endothelial cells.^[15]^ Therefore, if the 2 staining reagents are used together to determine the damaged area of corneal endothelial cells, both dead cells and healthy endothelial cells will be recognized. Alizarin red S, however, is only used in ex vivo experiments. This study was a retrospective study of corneal endothelial cell damage of donor corneas for corneal transplantation to patients, and it is not possible to use Alizarin red S in donor corneas.

In 3 eyes, the trypan blue staining was performed before scoring with a Sinskey. In 10 eyes, the trypan blue staining was performed after partial trephination for Descemet membrane peeling. In 8 eyes, the trypan blue staining was performed after scoring using a Sinskey hook for Descemet membrane peeling. The ideal way to measure corneal endothelial damage after only enucleation, buttoning, and storage is to perform trypan blue staining before scoring with a Sinskey hook or partial trephination. A few years ago, we stained endothelial cells with trypan blue, then scored Descemet membranes with a Sinskey hook, and stained endothelial cells with trypan blue again for Descemet membranes peeling. However, after we recognized that trypan blue staining before scoring with Sinskey hook does not help Descemet membranes peeling, we stained donor corneal endothelium only after Sinskey hook or partial trephination. This will reduce unnecessary endothelial cell damage caused by trypan blue staining. Therefore, for 3 eyes stained with trypan blue before membrane scoring, endothelial cell damage in the entire area would be more accurate than in the other eyes. In addition, since they were stained before and after Descemet membranes scoring, even minimal endothelial cell damage would be clearly visible.

For other 18 eyes, endothelial cell damage from scoring or partial trephination was not included in the trypan blue stained area because we wanted to investigate the endothelial cell damage resulting only from enucleation, corneal buttoning, and storage. In cases in which a Sinskey hook was used for scoring (8 eyes), it was often difficult to distinguish the trypan blue stained area induced by the scoring from the stained area caused by corneal buttoning. Therefore, when calculating the percentage of the stained area in the entire corneal endothelia, these 8 eyes were excluded from the analyses. In cases of partial trephination (10 eyes), endothelial cell damage from partial trephination was not included in the trypan blue stained area. Because of this, endothelial cell damages are likely to be slightly underestimated for these 10 eyes. However, for the percentage of the stained area in a circle with a diameter of 8 mm without the effect of Sinskey hook or partial trephination, there would be no such difference between 3 eyes and the remaining 18 eyes.

In conclusion, we quantitatively analyzed the corneal endothelial damage due to enucleation, storage, and buttoning by trypan blue staining for donor corneas that were transplanted into patients. The amount of corneal endothelial cell damage in the central 8-mm circle was mostly acceptable (3.4 ± 5.2%), but some cases showed significantly severe endothelial cell damage (10.5–17.8%). The central corneal thickness and endothelial cell density measured before corneal transplantation did not significantly correlate with the percentage of the stained area in the central 8-mm diameter circle. Therefore, pachymetry and specular microscopy are not sufficient for evaluating donor corneas before keratoplasty.

## Acknowledgments

Supported by a grant of the Korea Health Technology R&D Project through the Korea Health Industry Development Institute (KHIDI), funded by the Ministry of Health & Welfare, Republic of Korea (grant number: HI17C0659), Basic Science Research Program through the National Research Foundation of Korea (NRF), funded by the Ministry of Education, Republic of Korea (No. 2017R1A1A2A10000681, 2020R1A2C1005009)

## Author contributions

Conceptualization: Ho Sik Hwang.

Data curation: Young Chae Yoon, Yong-Soo Byun, Patrick Kim, Min Ji Ha.

Formal analysis: Young Chae Yoon.

Investigation: Young Chae Yoon, Yong-Soo Byun, Patrick Kim, Min Ji Ha.

Woong Joo Whang, Kyung Sun Na, Eun Chul Kim, Hyun Seung Kim, Ho Sik Hwang.

Methodology: Young Chae Yoon, Ho Sik Hwang.

Project administration: Young Chae Yoon, Ho Sik Hwang.

Resources: Young Chae Yoon.

Software: Young Chae Yoon.

Supervision: Woong Joo Whang, Kyung Sun Na, Eun Chul Kim, Hyun Seung Kim, Ho Sik Hwang

Validation: Young Chae Yoon, Ho Sik Hwang.

Visualization: Young Chae Yoon, Ho Sik Hwang.

Writing – original draft: Young Chae Yoon, Ho Sik Hwang.

Writing – review & editing: Woong Joo Whang, Kyung Sun Na, Eun Chul Kim, Hyun Seung Kim, Ho Sik Hwang.
